# Decreasing Social Support Associated With Risk of MCI and Dementia Is Partially Mediated by Hippocampal Volume

**DOI:** 10.1093/geroni/igaa057.1530

**Published:** 2020-12-16

**Authors:** Tara Gruenewald, Andrew Petkus, Xinhui Wang, Diana Youan, Margaret Gatz, Mark Espeland, Jiu-Chiuan Chen

**Affiliations:** 1 Chapman University, Orange, California, United States; 2 USC Keck School of Medicine, Los Angeles, California, United States; 3 University of Southern California, Los Angeles, California, United States; 4 Wake Forest School of Medicine, Winston-Salem, North Carolina, United States

## Abstract

Less supportive social relationships are linked to greater risk of cognitive decline in older adulthood. Few studies have examined if declines in social support predict risk of developing Mild Cognitive Impairment (MCI) or dementia and the neurobiological factors that may contribute to these associations. We analyzed data from 926 women in the Women’s Health Initiative Memory Study-MRI (WHIMS-MRI) to examine whether low social support at baseline and declines over an 8-year period predicted subsequent risk of developing MCI/dementia. Social support (Medical Outcomes Study Social Support Scale) was self-reported at the baseline (1994-1998) and closeout (2004-2005) of the parent WHI hormone therapy clinical trial. Annual neuropsychological assessments were conducted in WHIMS (through 2018) to ascertain incident MCI/dementia; structural brain scans were performed in 2005-2006. Structural equation models assessed the association between level and change in social support and risk of incident MCI/dementia and putative mediation of these associations by structural brain variables in women free of MCI/dementia as of the trial closeout, adjusting for demographic, lifestyle, depression, and biomedical covariates. Both low baseline social support (HR=1.24 per 1-SD; p<.05) and declines in support (HR=1.18 per 1-SD; p<.05) predicted incident MCI/dementia risk. Women reporting decreasing social support had significantly lower hippocampal volumes (β=-.070; p<.05) which accounted for ~14% of the total effect of declining support on MCI/dementia risk. We will highlight the implications of these findings for understanding how changes in social support may be linked to risk of MCI/dementia, including potential bidirectional associations of changes in social support and neurobiological health.

